# Supplementary material for: Spinful hinge states in the higher-order topological insulators WTe2
Source: Nat Commun. 2023 Mar 31;14:1801. doi: 10.1038/s41467-023-37482-0 (PMC10066182; doi:10.1038/s41467-023-37482-0)
Supplement: Supplementary file 1 — Supplementary Information [file 41467_2023_37482_MOESM1_ESM.docx]

# Supplementary Information for Spinful hinge states in the higher-order topological insulators WTe_2_

# Jekwan Lee^1,2^, Jaehyeon Kwon^1,2^, Eunho Lee^1,2^, Jiwon Park^1,2^, Soonyoung Cha^3,4^, Kenji Watanabe^5^, Takashi Taniguchi^5^, Moon-Ho Jo^3,4^, and Hyunyong Choi^1,2^

^1^Department of Physics and Astronomy, Seoul National University, Seoul 08826, Korea

^2^Institute of Applied Physics, Seoul National University, Seoul 08826, Korea

^3^Center for Epitaxial van der Waals Quantum Solids, Institute for Basic Science, Pohang 37673, Korea

^4^Department of Materials Science and Engineering, Pohang University of Science and Technology, Pohang 37673, Korea

^5^Advanced Materials Laboratory, National Institute for Materials Science, 1-1 Namiki, Tsukuba 305-0044, Japan

**Contents**

Supplementary Notes

1. Sample preparation 2

2. Experiment details 6

3. Extended dataset 9

4. Exclusion of the spin Hall effect 13

References 15

Supplementary Table 18

Supplementary Figures 19

**Supplementary Note 1 - Sample preparation**

1-1. Device fabrication

The devices used in this study were made of mechanically exfoliated flakes from single-crystal WTe_2_ (HQ Graphene) and single-crystal graphene (HQ Graphene). High-quality single-crystal hBN was provided by Advanced Materials Laboratory (National Institute for Materials Science, Japan). Poly(bisphenol A carbonate) (PC) coated polydimethylsiloxane (PDMS) was used to pick up hBN, graphene, and WTe_2_, and then they were transferred onto the pre-patterned electrodes^1,2^. All devices were fabricated based on the following procedure.

1. Si substrate (SiO_2_ 300 nm) cleaning

2. Bottom gate electrode patterning by e-beam lithography

3. Cleaning by O_2_ plasma using a reactive ion etcher (50 sccm, 5 seconds) after the development

4. Bottom gate electrode deposition by the thermal evaporator (Ti 5 nm, Au 25 nm)

5. Liftoff (warm acetone)

6. Gate insulator (hBN, 25 nm) transfer using PC pickup and stamping method

7. Electrical contact electrode fabrication (the same procedure used to fabricate the bottom gate, process 2 ~ 5)

8. Transfer monolayer graphene onto the device made up to this process

9. Pickup hBN and multilayer WTe_2_ using PC in order

10. Align and transfer the hBN and WTe_2_ onto the device made up to this process

11. Dissolve PC using Chloroform

Every exfoliation, pickup, and transfer process were proceeded inside a glovebox (N_2_ filled, O_2_ and H_2_O density of less than 0.5 ppm). A home-built transfer system in the glovebox is equipped with a hot chuck, a motorized rotation stage (PRMTZ8/M, Thorlabs), a piezo stage (MAX311D/M, Thorlabs), and a motorized stage (PLS-XY, Thorlabs), all of which was used for the precise transfer operation. The complete devices were stored in a high vacuum chamber for ~10 hours to achieve optimal electrical characteristics (i.e., reducing the contact resistance). Figure S1a shows the optical microscopy image of a complete device (device #1). The thickness of WTe_2_ was confirmed by atomic force microscopy (AFM), as shown in Fig. S1b. In Fig. S1c, we show the light polarization-dependent second harmonic generation (SHG) using a 30-fs, 800-nm short pulse (Mira 900, Coherent), where a typical measurement result of the multilayer WTe_2_ used in experiments is shown. The significant amount of SHG shows the lack of inversion symmetry in the multilayer WTe_2_. In addition, the anisotropic SHG reveals the axis orientation of the multilayer WTe_2_. The orientation is consistent with the result of the polarization-dependent absorption measurement (Fig. 1d in the main text)^3^.

1-2. Device characteristics (device #1)

The two-terminal I-V curve and *V*_G_-dependent transfer curve (at the bias voltage of 0.5 V) for device #1 are shown in Fig. S1d, e. All contacts exhibit an Ohmic junction (linear I-V curves). No significant variation of the resistance was observed (about 600 Ω) for different contact combinations. This corroborates that the contact quality of our device is uniform^4,5^. The transfer curve in Figs. S1e,f shows the effect of electrostatic doping on the current flow in our heterostructure. A charge neutral point of graphene was observed (blue down arrow), and the charge neutral point of the WTe_2_ was also observed when the current was measured between contact 1, 3 and contact 2, 4 (green down arrow), as shown in Fig. S1e. This provides evidence that the current flows through both graphene and WTe_2_.

We have estimated the Fermi-level change Δ*E*_F_ with respect to the applied bottom gate voltage *V*_G_, where Δ*E*_F_ is a differential change of *E*_F_ compared to *V*_G_ = 0 V, using a simple electrostatic calculation of $Q_{\text{2D}}=C_{\text{i}}V_{\text{i}}$ ^6^. Here, $Q_{\text{2D}}=eA\int_{0}^{E_{F}} \frac{2\left| E \right|}{\pi\hbar^{2}v_{F}^{2}}dE$ is the total charges accumulated in graphene with area *A* due to the gate voltage, $C_{i}=\varepsilon\frac{A}{d}$ is the capacitance of the graphene-hBN-electrode capacitor, and *V*_i_ is the gate voltage applied across the gate insulator hBN. The Fermi-level change Δ*E*_F_ was estimated using the following parameters: the Fermi velocity of graphene near Dirac point **_F_ ~ 1ⅹ10^6^ m/s ^7^, the permittivity of hBN *ε* = 3.75* ε*_0_ ^8^, and the thickness of hBN *d* = 25 nm (for device #1). From these values, the changes in Fermi level Δ*E*_F_ induced by the gate voltage *V*_G_ were estimated. For example, *V*_G_ of -1 and 2 V corresponds to -10 and 20 meV, respectively.

Figure S1g shows the magnetoresistance of the multilayer WTe_2_ without the graphene layer fabricated in a Hall bar geometry. The maximum magnetic field of our system (attoDRY 2100, Attocube systems) is 9 T. The non-saturating magnetoresistance up to 9 T can be attributed to the compensated electron and hole density^9^, proving the high quality of the WTe_2_ single crystal.

1-3. Additional devices

Additional devices were fabricated with the multilayer WTe_2_ of various thicknesses. The optical microscope images of all devices used in our study are shown in Fig. S2. Figure S3 shows the thickness of WTe_2_ for each device checked by the atomic force microscopy (AFM) measurements. Figure S4 shows the *V*_G_-dependent electrical characteristics of the devices. The device number and the associated parameters (thickness, number of layers, and charge-neutral *V*_G_, *V*_CNP_) are summarized in Table S1. Please note that we have acknowledged two recent studies by K. Kang et al. (Nat. Mater. (2019)) and Z. Fei et al. (Nature (2018)) to estimate the number of layers from the AFM measurements^10,11^. For the devices, device #1 is the one used to obtain Figs. 1-3 in the main text. Devices #2 and # 3 are the devices additionally fabricated to check the reproducibility. Device #4 is a device with a slightly different structure, where there is a “spatial gap” in graphene used to investigate the exclusive role of the hinge states on the Δ*θ*_K_ distribution (see Supplementary Note 4 for details).

**Supplementary Note 2 – Experiment details**

2-1. Experimental setup

In this study, the spatially resolved differential Kerr rotation (Δ*θ*_K_) measurement using a WTe_2_-graphene heterostructure device was designed for the detection of spin-polarized electrons in graphene that are injected from the multilayer WTe_2_. Figure S5 shows how our heterostructure device works. The electrodes are directly contacted with graphene only, thereby the heterostructure can be modeled as a resistive circuit with two resistors connected in a parallel way. When the bias voltage is applied to graphene, the current is induced in WTe_2_ as well as in graphene because there is finite resistance both for WTe_2_ and graphene channels. As a result, a portion of electrons in graphene flows through WTe_2_, and those electrons are injected back to graphene during the conduction. Therefore, we can detect the signature of the spin-polarized states in WTe_2_ by optically inspecting the spin-polarized carriers in graphene. To cancel out the effect of unintentional background factors, such as defects in graphene or chemical residues during the device fabrication process, the spatially resolved *θ*_K_ was measured with and without the bias voltage (*V*_bias_ = 0.5 V).

There are following technical reasons why we used graphene to detect the signature of the hinge states. First, the spatial length scale of the hinge states, whose width is only about a few nanometers^12,13^, is far smaller than the spot size of the incident laser beam (diameter ~1.5 μm). This means that any optical signals originating from the hinge states would be extremely weak compared to that from the bulk, surface, or other background signals generated within the beam spot. This scale issue limits the signal-to-noise ratio and hinders the direct detection of the hinge state if Δ*θ*_K_ were to be measured on the laser-excited hinges. Secondly, because the hinge, by definition, is a junction between two surfaces, an incident laser introduces both the in-plane and the out-of-plane excitation at the two surfaces facing the hinge. In addition, while the light excitation occurs on a 2D surface, the 1D hinge may induce an anisotropic polarization-dependent reflection of light, thereby preventing the clear isolation of Δ*θ*_K_ from the possible spin-polarized state.

To minimize the geometric effects of the hinge described above, we take advantage of graphene. Graphene is known to exhibit a long spin diffusion length of up to 30 μm due to the weak spin-orbit coupling and high mobility^14-16^. Under the electric field applied to the device, the electric potential gradient generates the longitudinal electron transport that injects electrons from WTe_2_ to graphene, as described in Supplementary Note 2-1. The spin polarization of the injected electrons can survive long enough to be detected by optical Kerr measurements due to the long spin diffusion length of graphene. Therefore, because Δ*θ*_K_ is distributed into the graphene rather than the localized WTe_2_ hinges, we have chosen a line cut at x = 0.75 μm to analyze Δ*θ*_K_ while keeping a certain distance from the edge of the WTe_2_ crystal.

2-2. Spatially resolved differential Kerr rotation measurements

The Kerr rotation measurements presented in the main text were performed at a temperature of 1.62 K in a closed-cycle cryostat system (attoDRY 2100, Attocube systems) combined with an objective lens (LT-APO/NIR/0.81, Attocube systems). The position of the sample was adjusted by piezo stages equipped inside the cryostat, and the position of the pump laser was controlled by a scanning mirror pair (GVS012/M, Thorlabs). The pump laser was obtained by using a 980 nm centered bandpass filter (FB980-10, Thorlabs) from the supercontinuum light source (SuperK COMPACT, NKT Photonics). The pump power at the sample position was 25 W, and the light polarization was tuned by using a polarizer (GL10-B, Thorlabs) and a /2 wave plate (AHWP05M-980) mounted on a motorized rotation stage (PR50PP, Newport). The pump laser was mechanically chopped with a frequency of 1.7 kHz. The Kerr rotation was measured using a Wollaston prism (WP10, Thorlabs) and a balanced detector (Nirvana model 2017, Newport) with a lock-in amplifier (SR830, Stanford Research Systems). The bias voltage and gate voltage was controlled by the electrical data acquisition system (BNC-2110 and DAQ, National Instruments). The bias voltage applied to the device during the experiment was 0.5 V.

**Supplementary Note 3 – Extended dataset**

3-1. Extended dataset (device #1)

In the main text, we presented the line-cut plots in Figs. 2 and 3 to show how Δ*θ*_K_ varies as a function of *V*_G_ and *B_z_*. In Figs. S6-9, full datasets obtained from the spatially resolved measurements on Δ*θ*_K_ for each case are presented. Figure S6 is the 2D contour plot of the *V*_G_-dependent Δ*θ*_K_ shown in Fig. 2. The monotonic decrease and the sign flip of Δ*θ*_K_ with varying *V*_G_ are clearly seen near the hinges when the Fermi level changes within the bulk bandgap (0 V ≤ *V*_G_ ≤ 1 V). The localized hinge Δ*θ*_K_ vanishes in a degenerately doped regime (*V*_G_ = -1.5, -1, 2, 3 V). The relatively weak Δ*θ*_K_ at |y| ≤ 1.85 μm originates from the gapped, spin-split bulk (or surface) states^17^. Figures. S7 and 8 are the same Δ*θ*_K_ data when *B_z_* is 0.5 T and 1 T, respectively. The results show that the hinge state disappears for 1 V ≤ *V*_G_ ≤ 1.1 V (Fig. S7) and for 0.8 V ≤ *V*_G_ ≤ 1.2 V (Fig. S8). In Fig. S9, the localized Δ*θ*_K_ is barely observed for a full range of *V*_G_ when *B_z_* is 2 T.

3-2. Bias voltage along *b*-axis (device #1)
 We have additionally measured the spatially resolved Δ*θ*_K_ with the bias voltage along the *b*-axis. Figure S10 shows Δ*θ*_K_ near the upper edge of WTe_2_ when the bias voltage is applied between contact 2 and 4, i.e., along the *b*-axis. Unlike the data shown in Figs. 2 and 3, no localized Δ*θ*_K_ is observed near the hinge at any *V*_G_. This absence of the localized Δ*θ*_K_ for electrons flowing along the *b*-axis suggests the anisotropic nature of the hinge state, as reported in prior investigations on multilayer WTe_2_^18,19^. It also provides the clue that the transverse spin current (i.e., spin Hall current) induced in WTe_2_ or graphene by the longitudinal field is irrelevant to the observed Δ*θ*_K_ features^20,21^. Besides, the transverse conductance due to SOC in graphene cannot explain the *V*_G_–dependent transition between the hinge- and the bulk-localized Δ*θ*_K_ as well.

3-3. Temperature-dependent Δ*θ*_K_ (device #1)

We also examine the temperature dependence of Δ*θ*_K_ from the hinge and the bulk to verify that the observed hinge state does not arise from the simple accumulation of carriers in the topologically trivial bulk or surface. Figure S11 shows the temperature dependence of Δ*θ*_K_ when *V*_G_ = 0 (a) and 2 V (b). To compare Δ*θ*_K_ from the hinge and bulk of WTe_2_, |Δ*θ*_K_| was averaged (|Δ*θ*_K_|_avg_) in the specific region near the hinge and bulk (dashed boxes in Fig. S9). Figure S12 shows the summarized temperature dependence of |Δ*θ*_K_|_avg_. |Δ*θ*_K_|_avg_ near the hinge drops rapidly below the noise level as the temperature rises and becomes negligible when the temperature is higher than 40 K; while |Δ*θ*_K_|_avg_ near the bulk remains relatively unchanged.
The distinction of the temperature dependence of |Δ*θ*_K_|_avg_ between the hinge and the bulk reveals that the origin of the spinful hinge states differs from the trivial spin-split bulk band or 2DEG^22,23^. In detail, the temperature dependence of |Δ*θ*_K_|_avg_ from the hinge, i.e., across 40 K, can be related to a transition from the topological hinge to the trivial semi-metallic phase^24^, while |Δ*θ*_K_|_avg_ from the bulk is barely changed with increasing the temperature.

3-4. Spatially resolved *V*_G_-dependent Δ*θ*_K_ from additional devices (devices #2 and #3)

Figure S13 shows the spatially resolved Δ*θ*_K_ of devices #2 (Fig. S13a) and #3 (Fig. S13b) with varying *V*_G_ across the Dirac point (see Fig. S4 for the *V*_G_-dependent transfer characteristics for each device). For device #1, the measured data are shown in Figs. 2 and 3 and also in Figs. S4-9. For device #4, the associated contents are discussed in Supplementary Note 4. All the measurements were performed under the same condition as the experiments performed with device #1 (e.g., cryogenic condition, chopping frequency, pump energy and power, bias voltage). From Fig. S13, we see that Δ*θ*_K_ is localized at *y*-positions of the WTe_2_ hinges, and its sign is flipped as *V*_G_ passes across *V*_CNP_. These features are identical to the *V*_G_-dependent distribution of Δ*θ*_K_ observed in device #1.

There are some details on the thickness-dependent characteristics that should be addressed. First, the multilayer WTe_2_ has been considered to be a type-II Weyl semimetal (WSM)^25^. The characteristic features of WSM are the existence of the Weyl point pair and the associated Fermi arcs connecting the two Weyl points. Investigation using surface-sensitive probes such as angle-resolved photoemission spectroscopy (ARPES) or scanning tunneling microscopy is a method to prove the WSM nature^26,27^. Another is to see the “chiral anomaly”, known as the Adler-Bell-Jackiw anomaly in high-energy physics^28,29^, by performing magneto transport experiments under parallel electric- and magnetic-field excitation, i.e., applying **E**∙**B**^30-32^. Others include measurements of the quantum critical resistivity, showing a temperature-dependent inverse power law of resistivity^33^; though this is not direct proof of the WSM nature. Unlike TaAs, TaP, and NbAs, it has not been clearly resolved the Weyl points and the Fermi arc in WTe_2_, and the experimental evidence so far is not quite conclusive^18,19,34,35^. In our study, Δ*θ*_K_ near the hinge-localized states is not related to the above signatures of WSM, while the measurements show the conjectures of spinful HOTI as predicted in theoretical works and recent experiments in other HOTI materials^36-38^.

Second, in the monolayer limit of 1T′-WTe_2_, the 2D quantum spin Hall insulator (QSHI) is now well established^39,40^. Further studies reveal that a bilayer WTe_2_ is topologically trivial and centrosymmetric^12,41,42^. Because our sample contains more than three layers, the multilayer WTe_2_ is no longer considered to be a 2D system. If our sample were to exhibit a 3D TI phase, then the surface should host topologically protected gapless states. Searching for existing ARPES literature, we were not able to find any such evidence^17,26^.

Lastly, a lower threshold of the layer numbers has been reported for *T*_d_-WTe_2_ to be a 3D HOTI^18,36^, which dictates that it should be at least three, while no theoretical or experimental study has reported the upper limit of the thickness of *T*_d_-WTe_2_ that guarantees the existence of the HOTI phase. For example, a recent study has shown that even a 20-nm-thick WTe_2_ (~ 25 atomic layers) can host a conducting channel localized at the hinge^18^. We expect that further theoretical studies are required to perform to determine the exact crossover of layer numbers and the upper bound for HOTI to occur.

**Supplementary Note 4 –Exclusion of the spin Hall effect**

As described in the main text (the related contents are shown in Fig. 4 in the main text), we experimentally show that the spin Hall effect (SHE) is not the major origin of Δ*θ*_K_ distribution. Generally speaking, the anomalous Hall effect arises from the bulk or the surface due to the Berry curvature or scattering under spin-orbit coupled magnetization^43^. It generates the transport of electrons transverse to the applied electric field, and more specifically, the transverse spin current can be induced by SHE. If SHE in the multilayer WTe_2_ is the alternative origin of the observed Δ*θ*_K_, then Δ*θ*_K_ distribution should appear as Fig. 4c in the main text. However, the data shown in Figs. 4d,e indicate that this is not the case. Figure S15 shows the full *V*_G_-dependent spatial distribution of Δ*θ*_K_ in device #4 with (Fig. S15a) and without (Fig. S15b) the external magnetic field. The line-cut plots in Fig. S16 show the *V*_G_- and *B_z_*-dependent Δ*θ*_K_ in device #4, which is identical to the *V*_G_ and *B_z_* dependence of Δ*θ*_K_ in device #1 shown in Fig. 3.

The Berry curvature dipole of WTe_2_ can also be considered as a possible origin of the observed Δ*θ*_K_. Here, the way how the Berry curvature dipole affects the transverse electrical transport is different from SHE. Recent studies on the highly asymmetric WTe_2_ revealed that the Berry curvature dipole is related to the nonlinear Hall effect in a non-magnetic environment^10,42,44^. The nonlinear Hall current $\vec{j}^{2\omega}$ can be written as

$$\vec{j}^{\omega}=\frac{e^{3}\tau}{2\left( 1+i\omega\tau\right)}\hat{z}\times\vec{E}\left( \vec{\Lambda}\cdot\vec{E} \right),$$

where *ω* is the frequency, *τ* is the Boltzmann transport relaxation time, and ** is the Berry curvature dipole^45^. Here, the nonlinear Hall effect induced by the Berry curvature dipole is a nonlinear response with respect to the applied sinusoidal electric field, and an electrical Hall effect causes a transverse charge current rather than the transverse spin current. Hence, Δ*θ*_K_ distributions observed in our experiments are not related to the nonlinear Hall effect by the Berry curvature dipole, even though the anisotropic Δ*θ*_K_ distribution matches that of the nonlinear Hall current. Moreover, even if the spin degree of freedom is assigned to the nonlinear Hall current for some reason, the nonlinear Hall effect should arise from the whole WTe_2_ area, not exclusively from the hinge.

**References**

1. Wang, L. *et al*., One-dimensional electrical contact to a two-dimensional material. Science **342**, 614-617 (2013).39
2. Wood, J. D. et al., Annealing free, clean graphene transfer using alternative polymer scaffolds. Nanotechnol. 26, 055302 (2015).
3. Tiwari, A. et al., Giant c-axis nonlinear anomalous Hall effect in Td¬-MoTe2 and WTe2. Nat. Commun. 12, 2049 (2021).
4. Fiori, G. et al., Electronics based on two-dimensional materials. Nat. Nanotechnol. 9, 768-779 (2014).
5. Cusati, T. *et al.*, Electrical properties of graphene-metal contacts. *Sci. Rep.* **7**, 5109 (2017).
6. Xu, S.-Y. *et al*., Electrically switchable Berry curvature dipole in the monolayer topological insulator WTe_2_. *Nat. Phys.* **14**, 900-906 (2018).
7. Bolotin, K. I., *et al*., Temperature-dependent transport in suspended graphene. *Phys. Rev. Lett.* **101**, 096802 (2008).
8. Laturia, A. *et al*., Dielectric properties of hexagonal boron nitride and transition metal dichalcogenides: from monolayer to bulk. *NPJ 2D Mater. Appl.* **2**, 6 (2018).
9. Wang, Y. *et al.*, Direct evidence for charge compensation-induced large magnetoresistance in thin WTe_2_. *Nano Lett.* **19**, 3969-3975 (2019).
10. Kang, K. *et al.*, Nonlinear anomalous Hall effect in few-layer WTe_2_. *Nat. Mater*. **18**, 324-328 (2019).
11. Fei, Z. *et al*., Ferroelectric switching of a two-dimensional metal. *Nature* **560**, 336-339 (2018).
12. Zheng, F. *et al*., On the quantum spin Hall gap of monolayer 1T′-WTe_2_. *Adv. Mater.* **28**, 4845-4851 (2016).
13. Schindler, F. *et al*., Higher-order topology in bismuth. *Nat. Phys.* **14**, 918-924 (2018).
14. Drögeler, M. *et al*., Spin lifetimes exceeding 12 ns in graphene nonlocal spin valve devices. *Nano Lett.* **16,** 3533-3539 (2016).
15. Kamalakar, M. V., *et al*., Long distance spin communication in chemical vapour deposited graphene. *Nat. Commun*. **6**, 6766 (2015).
16. Han, W., Kawakami, R. K., Gmitra, M., and Fabian, J., *et al*., Graphene spingronics. *Nat. Nanotechnol.* **9**, 794-807 (2014).
17. Das, P. K. *et al*., Layer-dependent quantum cooperation of electron and hole states in the anomalous semimetal WTe_2_. *Nat. Commun.* **7**, 10847 (2016).
18. Choi, Y.-B. *et al*., Evidence of higher-order topology in multilayer WTe_2_ from Josephson coupling through anisotropic hinge states. *Nat. Mater.* **19**, 974-979 (2020).
19. Kononov, A. *et al*., One-dimensional edge transport in few-layer WTe_2_. *Nano Lett.* **20**, 4228-4233 (2020).
20. Powalla, L. *et al*., Berry curvature-induced local spin polarization in gated graphene/WTe_2_ heterostructures. *Nat. Commun.* **13**, 3152 (2022).
21. Cui, J. *et al*., Transport evidence of asymmetric spin-orbit coupling in few-layer superconducting 1*T*_d_-MoTe_2_. *Nat. Commun*. **10**, 2044 (2019).
22. Yu, J. *et al.*, Photoinduced inverse spin Hall effect of surface states in the topological insulator Bi_2_Se_3_. *Nano Lett*. **17**, 7878-7885 (2017).
23. Thoutam, L. R. *et al.*, Temperature-dependent three-dimensional anisotropy of the magnetoresistance in WTe_2_. *Phys. Rev. Lett*. **115**, 046602 (2015).
24. Wu, S. *et al.*, Observation of the quantum spin Hall effect up to 100 kelvin in a monolayer crystal. *Science* **359**, 76-79 (2018).
25. Soluyanov, A. A., *et al*., Type-II Weyl semimetals. *Nature* **527**, 495-498 (2015).
26. Bruno, F. Y., *et al*., Observation of large topologically trivial Fermi arcs in the candidate type-II Weyl semimetal WTe_2_. *Phys. Rev. B* **94**, 121112(R) (2016).
27. Feng, B., *et al*., Spin texture in type-II Weyl semimetal WTe_2_. *Phys. Rev. B* **94**, 195134 (2016).
28. Adler, S. L., Axial-vector vertex in spinor electrodynamics. *Phys. Rev.* **177**, 2426 (1969).
29. Bell, J. S., and Jackiw, R. C. A PCAC puzzle: π^O^→ γγ in the σ-model. *Nuovo Cimento A* **60**, 47-61 (1969).
30. Xiong, J. *et al*., Evidence for the chiral anomaly in the Dirac semimetal Na_3_Bi. *Science* **350**, 6259 (2015).
31. Rylands, C. *et al*., Chiral anomaly in interacting condensed matter systems. *Phys. Rev. Lett.* **126**, 185303 (2021).
32. Ong, N. P. and Liang, S., Experimental signatures of the chiral anomaly in Dirac-Weyl semimetals. *Nat. Rev. Phys.* **3**, 394-404 (2021).
33. P. Telang, K. Mishra, G. Prando, A. K. Sood, and S. Singh, Anomalous lattice contraction and emergent electronic phases in Bi-doped Eu_2_Ir_2_O_7_. *Phys. Rev. B* **99**, 201112(R) (2019).
34. Xu, S.-Y. *et al*., Experimental discovery of a topological Weyl semimetal state in TaP. *Sci. Adv.* **1**, e1501092 (2015).
35. Yuan, X., *et al*., The discovery of dynamic chiral anomaly in a Weyl semimetal NbAs. *Nat. Commun.* **11**, 1259 (2020).
36. Wang, Z., *et al*., Higher-order topology, monopole nodal lines, and the origin of large Fermi arcs in transition metal dichalcogenides *X*Te_2_ (*X* = Mo, W). *Phys. Rev. Lett.* **123**, 186401 (2019).
37. Shumiya, N., *et al*., Evidence of a room-temperature quantum spin Hall edge state in a higher-order topological insulator. *Nat. Mater.* preprint (2022).
38. Noguchi, R., *et al*., Evidence for a higher-order topological insulator in a three-dimensional material built from van der Waals stacking of bismuth-halide chains. *Nat. Mater.* **20**, 473-479 (2021).
39. Fei, Z., *et al*., Edge conductio in monolayer WTe_2_. *Nat. Phys.* **13**, 677-682 (2017).
40. Tang, S., *et al*., Quantum spin Hall state in monolayer 1T′-WTe_2_. *Nat. Phys.* **13**, 683-687 (2017).
41. Lüpke, F., *et al*., Quantum spin Hall edge states and interlayer coupling in twisted bilayer WTe_2_. *Nano Lett.* **22**, 5674-5680 (2022).
42. Ma, Q., *et al*., Observation of the nonlinear Hall effect under time-reversal-symmetric conditions. *Nature* **565**, 337-342 (2019).
43. Nagaosa, N. *et al*., Anomalous Hall effect. *Rev. Mod. Phys.* **82**, 1539 (2010).
44. Du, Z. Z., Lu, H.-Z., and Xie, X. C., Nonlinear Hall effects. *Nat. Rev. Phys.* **3**, 744-752 (2021).
45. Sodemann, I. and Fu, L., Quantum nonlinear Hall effect induced by Berry curvature dipole in time-reversal invariant materials. *Phys. Rev. Lett.* **115**, 216806 (2015).

**Supplementary Table**

Table S1. Thickness and *V*_CNP_ of each device used in the experiments. The thickness was measured by AFM (Fig. S3), and *V*_CNP_ was obtained from the *V*_G_-dependent electrical transport (Fig. S4).

| Device | WTe_2_ thickness (nm) | Number of layers | *V*_CNP_(V) |
| --- | --- | --- | --- |
| device #1 | 4 | 5 | 0.95 |
| device #2 | 16 | ~20 | 1 |
| device #3 | 3 | 4 | 0.92 |
| device #4 | 5 | 7 | 0.88 |

**Supplementary Figures**


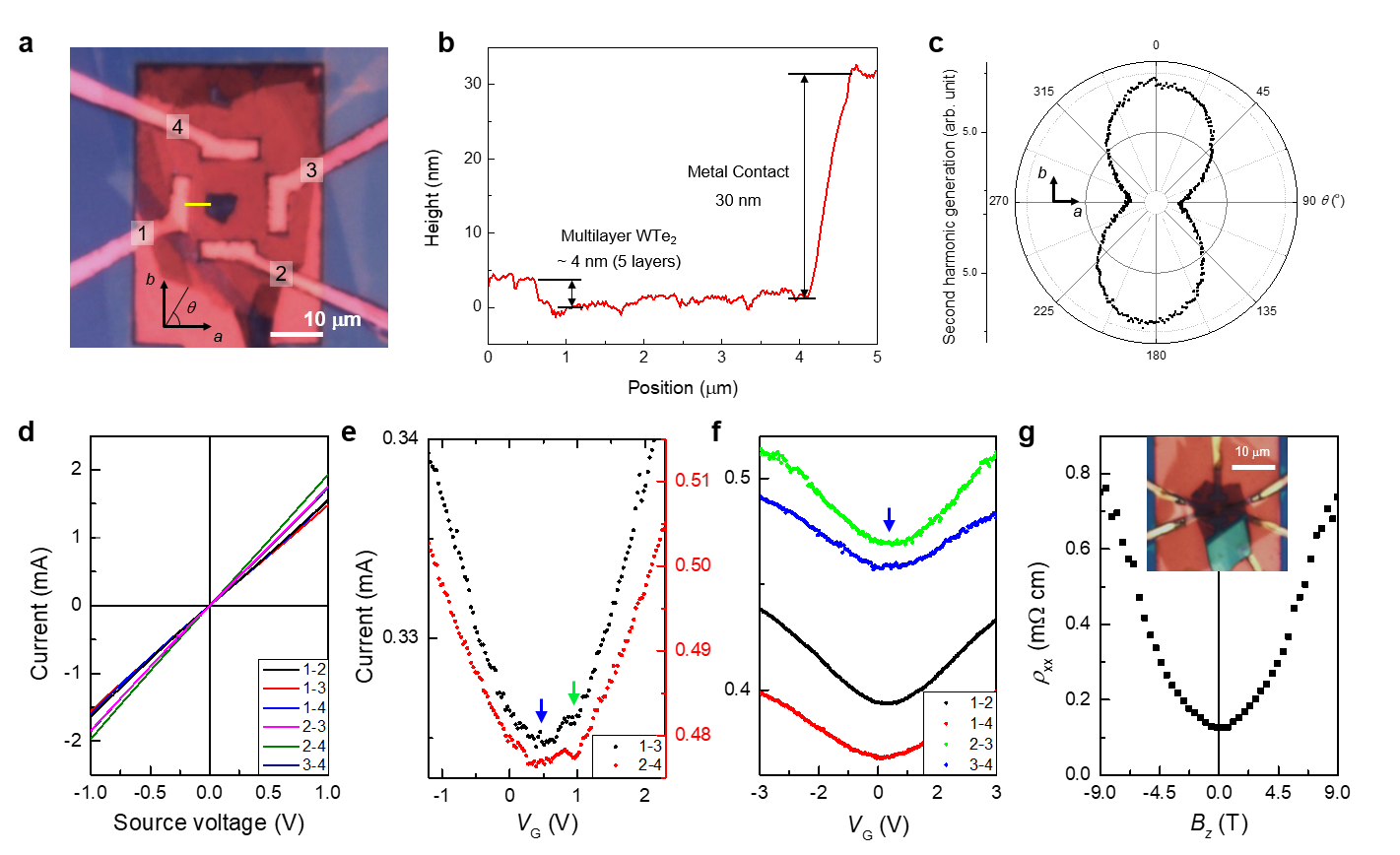


**Figure S1. Device geometry and electrical characteristics.** **a.** An optical microscope image of device #1 with the number index for each metal contact. We have taken the AFM scan along the yellow line. **b.** The AFM data indicate that the thickness of the multilayer WTe_2_ flake is ~ 4 nm, corresponding to 5 atomic layers of WTe_2_. The thickness of the metal contact shown in the data (30 nm) matches well with the targeted deposition thickness. **c.** The polar plot of the light polarization-dependent SHG measured at the center of the WTe_2_ flake in device #1. An 800 nm short pulse with an average intensity of 2,830 W/cm^2^ was used as a pump. **d.** Current-voltage output curve of device #1 for each pair of source and drain contact. All contacts form an Ohmic junction with graphene. Numbers in the legend indicate the contact combinations used in each measurement. **e,f**. *V*_G_-dependent transfer curve. The applied drain voltage was 0.5 V for the measurements. The curve index of 1-3 corresponds to the transfer curve shown in Fig. 2a in the main text. **g.** The magnetoresistance of the multilayer WTe_2_ was measured in a Hall bar geometry. The magnetoresistance does not saturate within |*B_z_*| ≤ 9 T. The applied gate voltage was 0.9 V, which is close to the charge neutral point. Inset shows the fabricated Hall bar device with the bottom gate electrode.


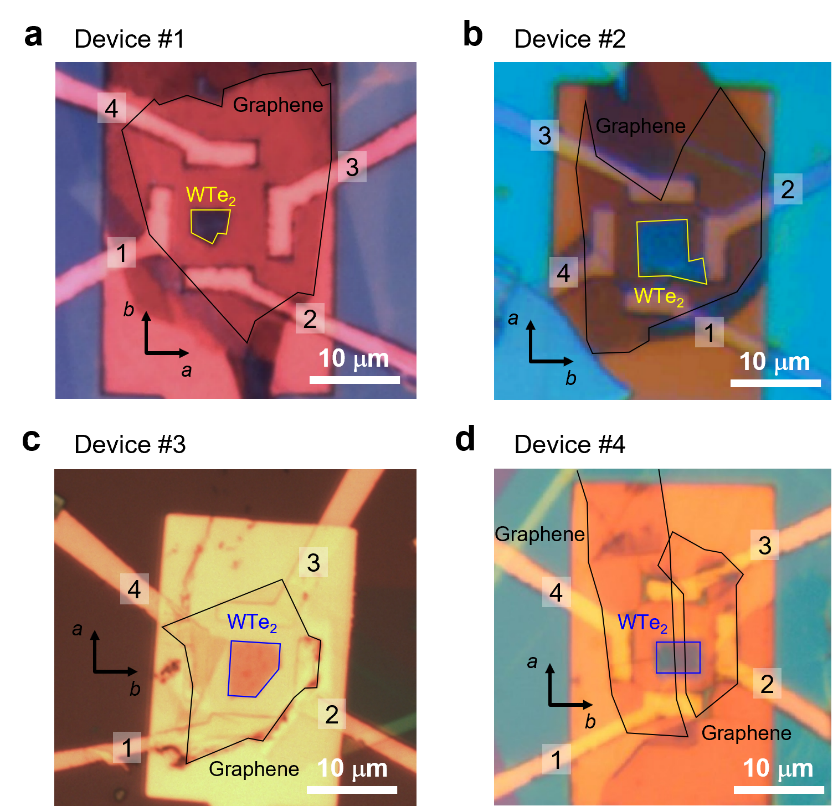


**Figure S2.** **Optical microscope images of all devices.** **a.** Device #1 is used to obtain the data in Figs. 1-3 in the main text. **b, c.** Devices #2 and #3 are additional devices with the same device structure as device #1. **d.** Device #4 has a 1.5 μm wide gap in the middle of graphene. All electrodes in the devices were made using 5 nm Ti and 25 nm Au. Numbers 1~4 in the figures are the contact indices. The black arrows in each figure show the orientation of WTe_2_ crystal axes.


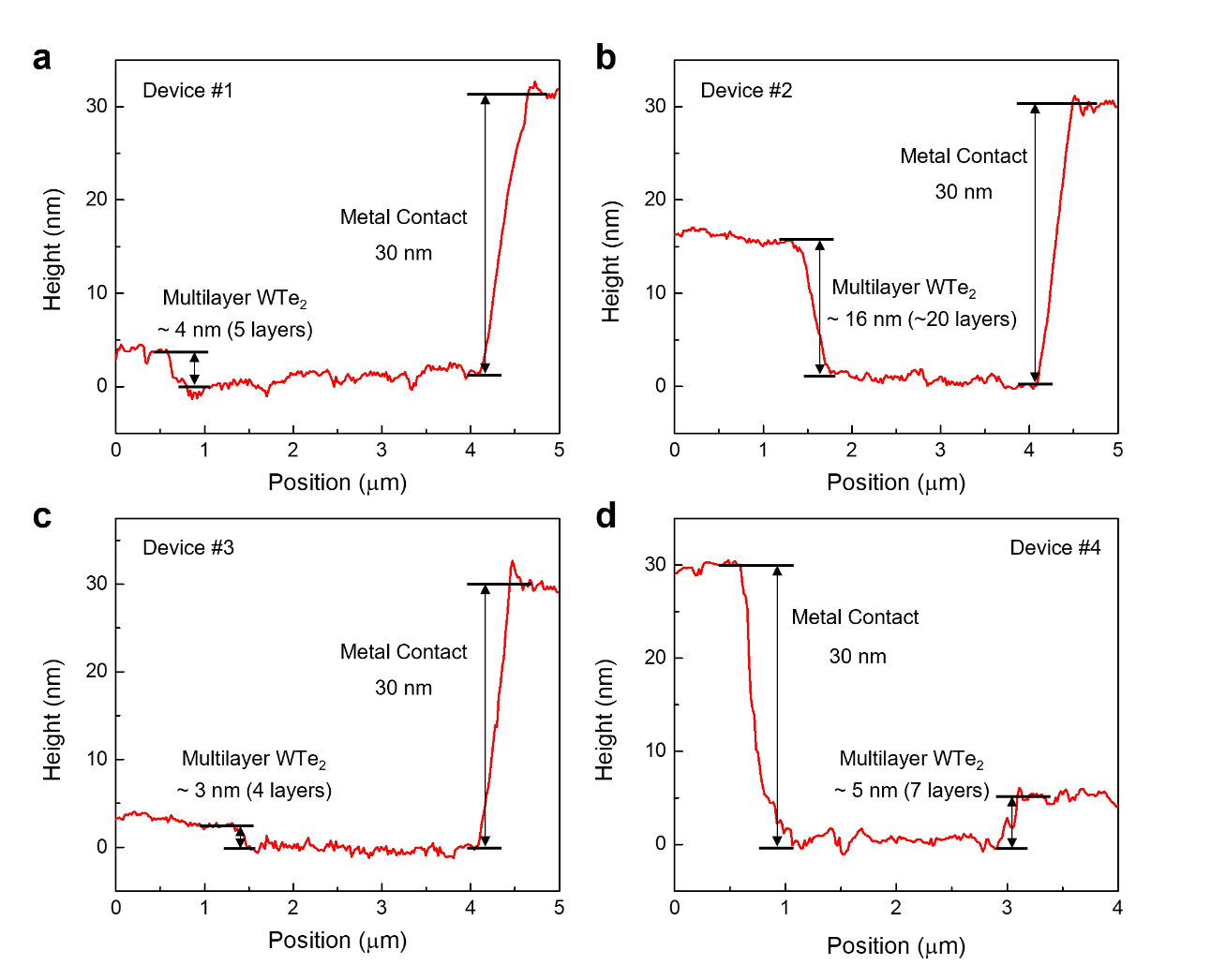


**Figure S3.** **AFM data.** **a-d.** The thickness of the multilayer WTe_2_ flake in the devices was measured by AFM. The thickness of the metal contact (Ti 5 nm, Au 25 nm) close to WTe_2_ is also measured for reference.


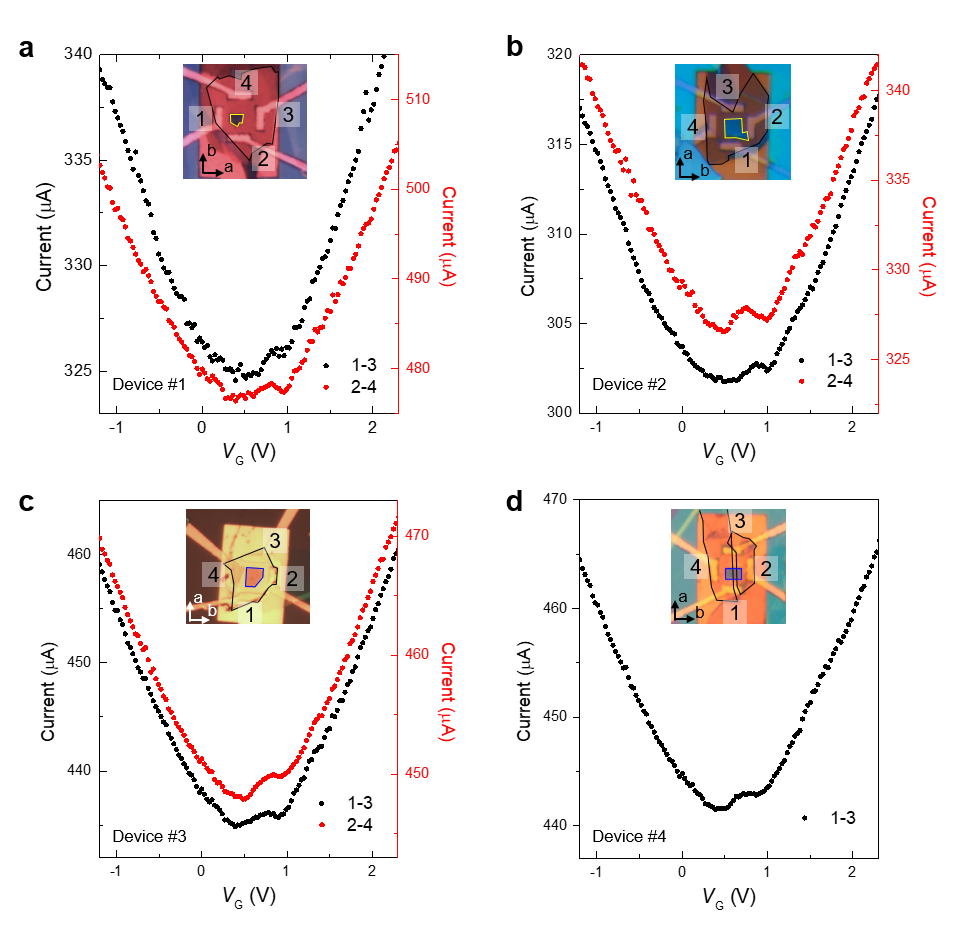


**Figure S4.** ***V*_G_**-**dependent electrical characteristics obtained from devices #1~4.** **a-d.** Indices 1-3 and 2-4 stand for the source and drain contact for each device. The current between contact 1, 3 (2, 4) corresponds to the current along the a- (b-) axis of each device.


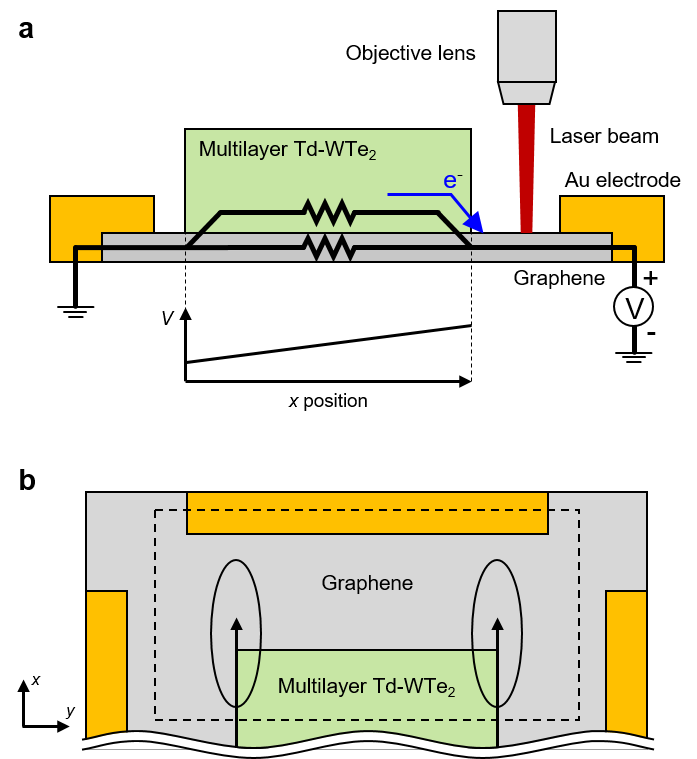


**Figure S5.** **Schematic diagram of the heterostructure device.** **a.** The WTe_2_-graphene hetero-structure can be modeled as a circuit with two finite resistance connected in a parallel way. The bias voltage applied between two contacts induces a current both through the WTe_2_ and the graphene channel. Electrons injected into graphene from WTe_2_ (blue arrow) are optically investigated to determine the spinful characteristics of the electrons in WTe_2_. **b.** Top view schematic diagram of the device explaining the region of the spatially resolved Kerr measurement. Dashed box indicates the 2D scanned spatial range under the longitudinal electric field –*y* direction. Arrow denotes the injection of electrons from WTe_2_ to graphene. The localized Δ*θ*_K_ was expected to appear in the region marked with black circles.


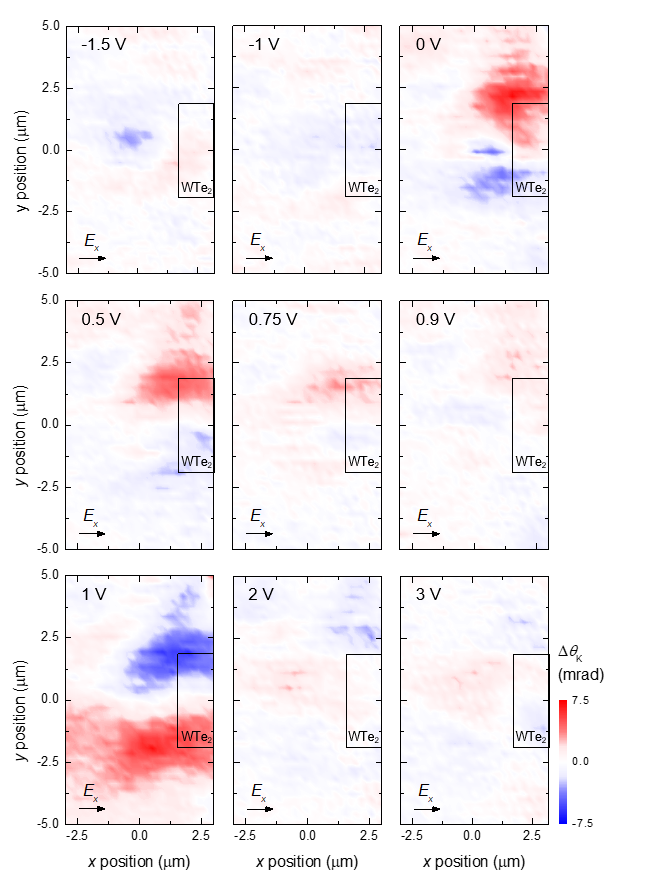


**Figure S6.** **The full dataset of spatially resolved *V*_G_-dependent Δ*θ*_K_ measurement (device #1).** 2D contour plots of the *V*_G_-dependent Δ*θ*_K_ are shown. No external magnetic field was applied. The bias voltage of 0.5 V was applied to generate a longitudinal electric field in +*x* direction. The applied *V*_G_ is indicated at the top left side of each contour plot. The black rectangle indicates the location of the left end part of the multilayer WTe_2_. When *V*_G_ is between 0 and 1 V, a large magnitude of Δ*θ*_K_ is visible near the hinges, as well as a sign flip of Δ*θ*_K_ near each hinge. We attribute these phenomena to the existence of spin-polarized states of the hinges.


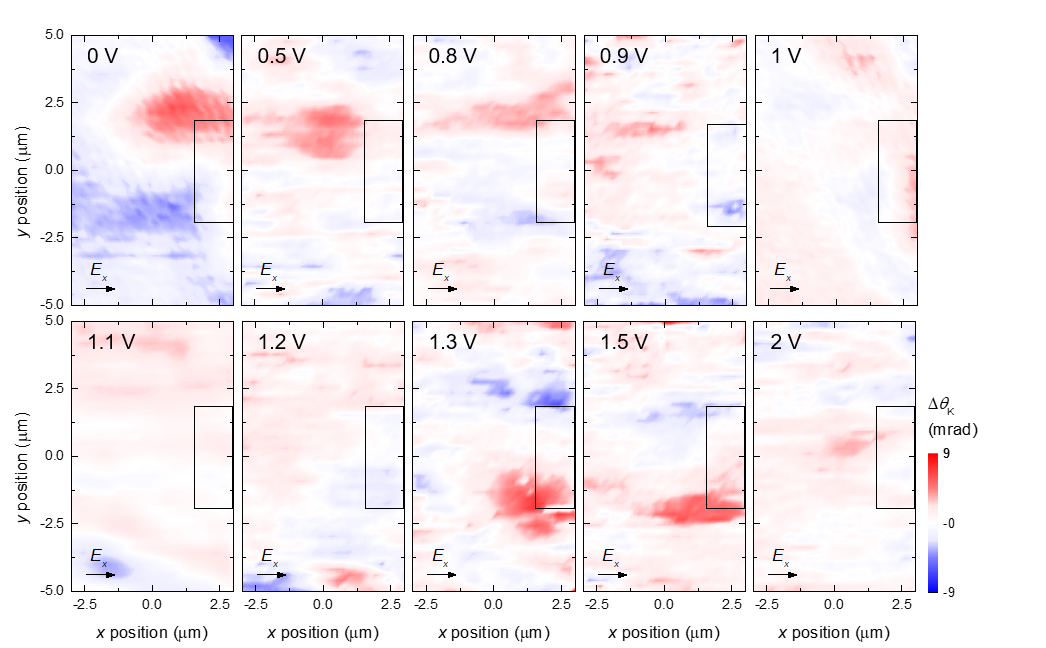


**Figure S7.** **The full dataset of spatially resolved *V*_G_-dependent Δ*θ*_K_ measured with *B_z_* = 0.5 T (device #1)**. 2D contour plots of the *V*_G_-dependent Δ*θ*_K_ are shown. The applied external magnetic field was 0.5 T. The bias voltage of 0.5 V was applied to generate a longitudinal electric field in +*x* direction. The applied *V*_G_ is indicated at the top left side of each contour plot. The black rectangle indicates the location of the left end part of the multilayer WTe_2_. Although the data are similar to Fig. S4, i.e., the *V*_G_-dependent sign flip of Δ*θ*_K_, the hinge-concentrated Δ*θ*_K_ disappears when *V*_G_ is near the charge neutral point (*V*_G_ = 0.95 V).


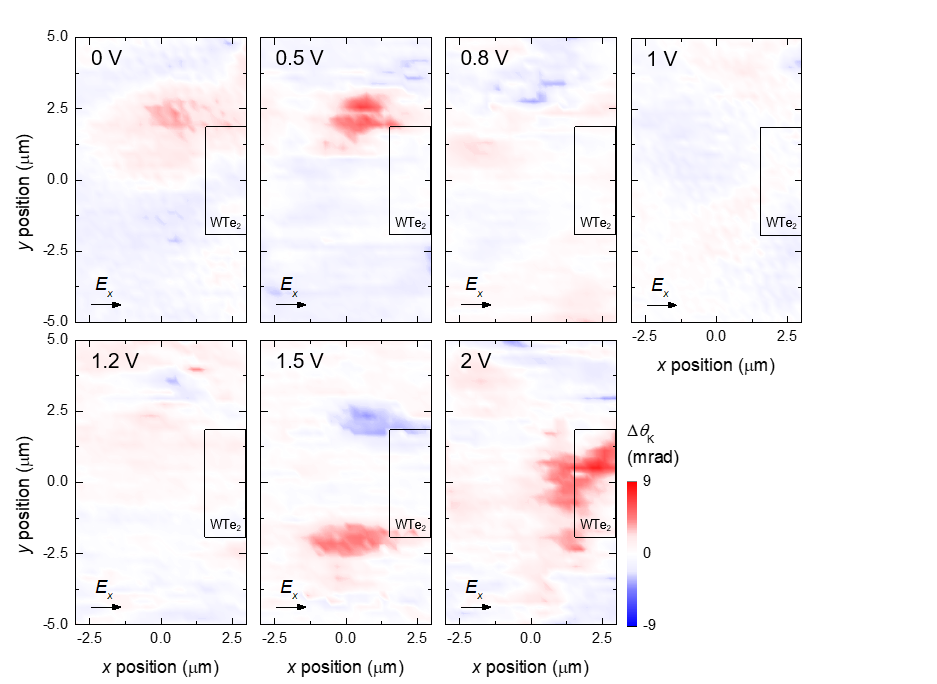


**Figure S8. The full dataset of spatially resolved *V*_G_-dependent Δ*θ*_K_ measured with *B_z_* = 1 T (device #1)**. 2D contour plots of the *V*_G_-dependent Δ*θ*_K_ are shown. The applied external magnetic field was 1 T. The bias voltage of 0.5 V was applied to generate a longitudinal electric field in +*x* direction. The applied *V*_G_ is indicated at the top left side of each contour plot. The black rectangle indicates the location of the left end part of the multilayer WTe_2_. No clear signal of hinge-concentrated Δ*θ*_K_ is visible when *V*_G_ is near the charge neutral point.

[영어로 어떤 뜻을 나타내는지 이해하기 어려워서 삭제했다]


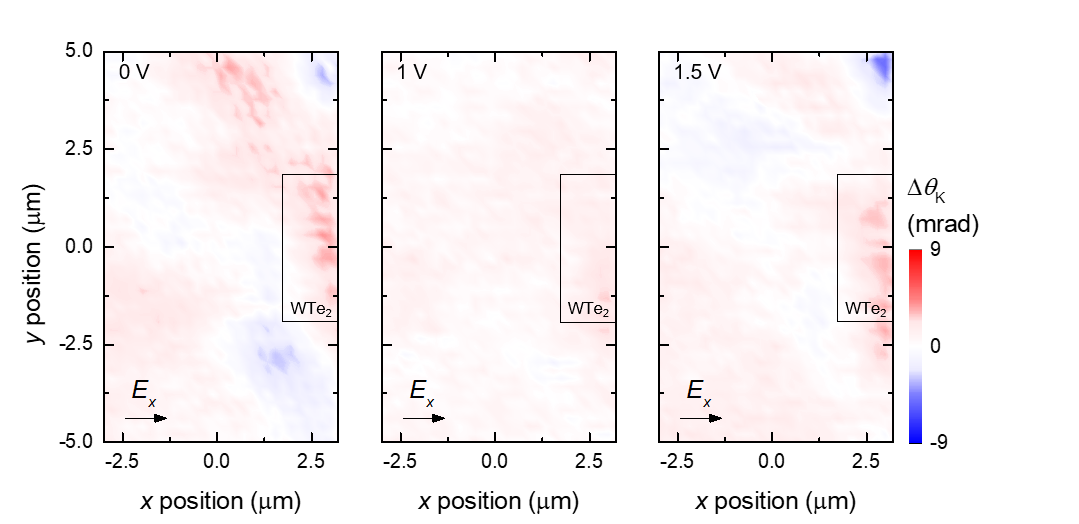


**Figure S9.** **The full dataset of spatially resolved *V*_G_-dependent Δ*θ*_K_ measured with *B_z_* = 2 T (device #1)**. 2D contour plots of the *V*_G_-dependent Δ*θ*_K_ are shown. The applied external magnetic field was 2 T. The bias voltage of 0.5 V was applied to generate a longitudinal electric field in +*x* direction. The applied *V*_G_ is indicated at the top left side of each contour plot. The black rectangle indicates the location of the left end part of the multilayer WTe_2_. The localized Δ*θ*_K_ completely disappears because of the broken time-reversal symmetry.


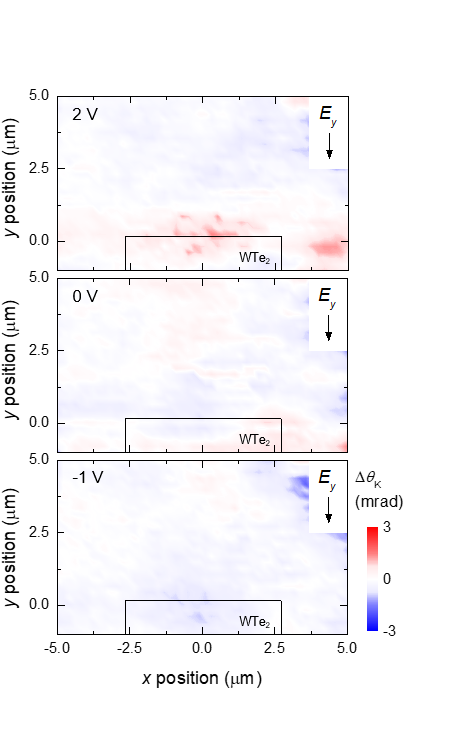


**Figure S10.** **Investigation into the hinges along *b*-axis.** Spatially resolved *V*_G_-dependent Δ*θ*_K_ when the bias voltage was applied along *b*-axis. Contour plots show the spatially resolved Δ*θ*_K_ near the top end part of the WTe_2_ flake (black rectangle). The electrical bias voltage was applied between contact 2 and 4 (parallel to the *b*-axis; see Fig. 1c for the contact number) with the applied bias of 0.5 V; the electric field direction is along –*y* direction. *B_z_* was set to be zero.


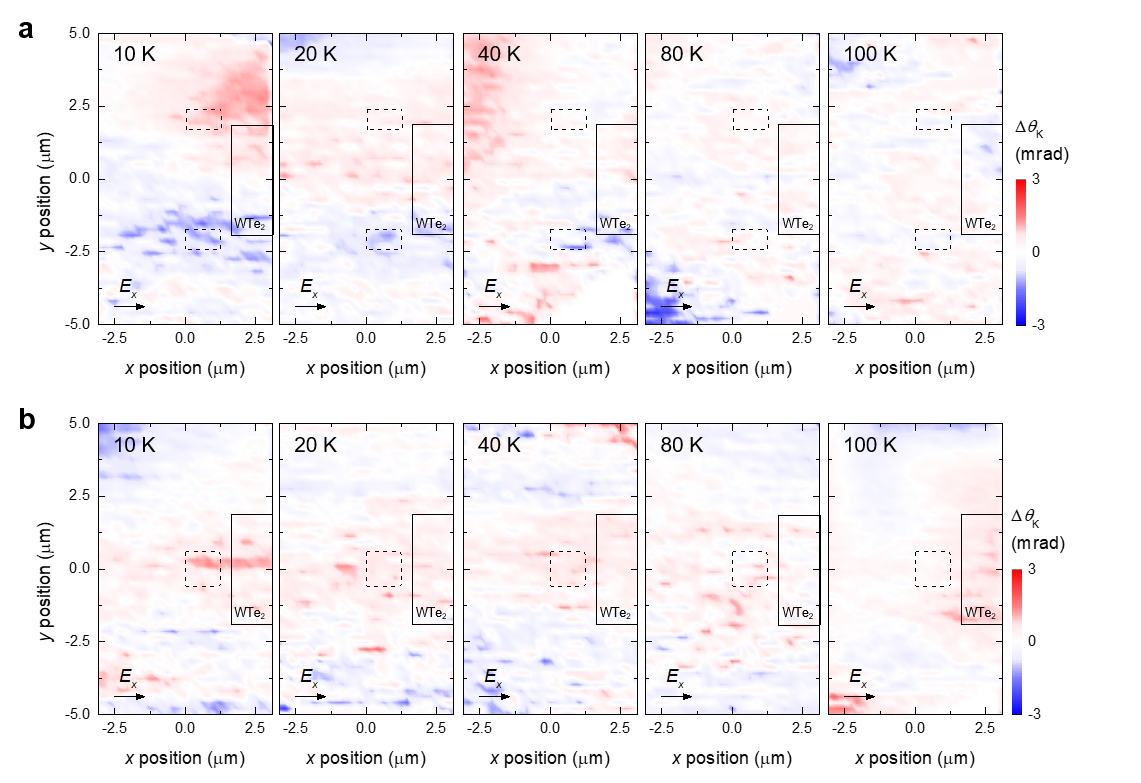


**Figure S11.** **The full dataset of temperature-dependent Δ*θ*_K_ measurement (device #1).** 2D contour plots of the temperature-dependent Δ*θ*_K_ with 0.5 V bias voltage (longitudinal field in +*x* direction) when *V*_G_ is 0 V (a) and 2 V (b). No magnetic field was applied. The temperature is indicated at the top left side of each contour plot. The black solid rectangle indicates the location of the left end part of WTe_2_. Dashed rectangles indicate the area where the average of |Δ*θ*_K_| was taken. Corresponding spatial ranges are 0 μm < *x* < 1.25 μm, 1.7 μm< |*y*| < 2.3 μm for **a**, and 0 μm < *x* < 1.25 μm, |*y*| < 0.6 μm for **b**.


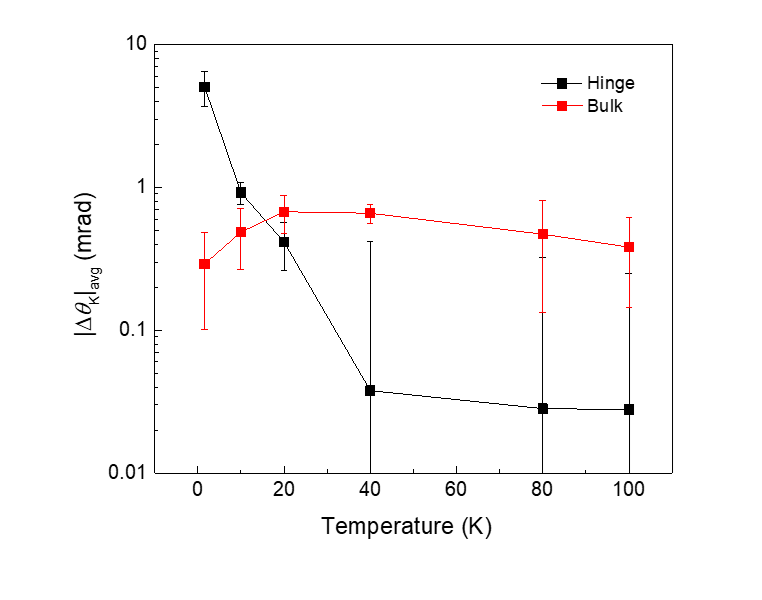


**Figure S12.** **Comparison of temperature dependent Δ*θ*_K_ between the hinge and the bulk.** The plot shows the temperature dependence of the average differential Kerr rotation |Δ*θ*_K_|_avg_ for the hinge (black) and bulk (red). The bias voltage was applied between contact 1 and 3 (parallel to the *a*-axis; see Fig. 1c for the contact index), and the scanned spatial range was near the left edge of WTe_2_, as shown in Fig. S9. The error bars indicate the standard deviation. Note |Δ*θ*_K_|_avg_ from the hinge drops drastically as the temperature increases, while |Δ*θ*_K_|_avg_ from the bulk is barely affected regardless of the temperature.


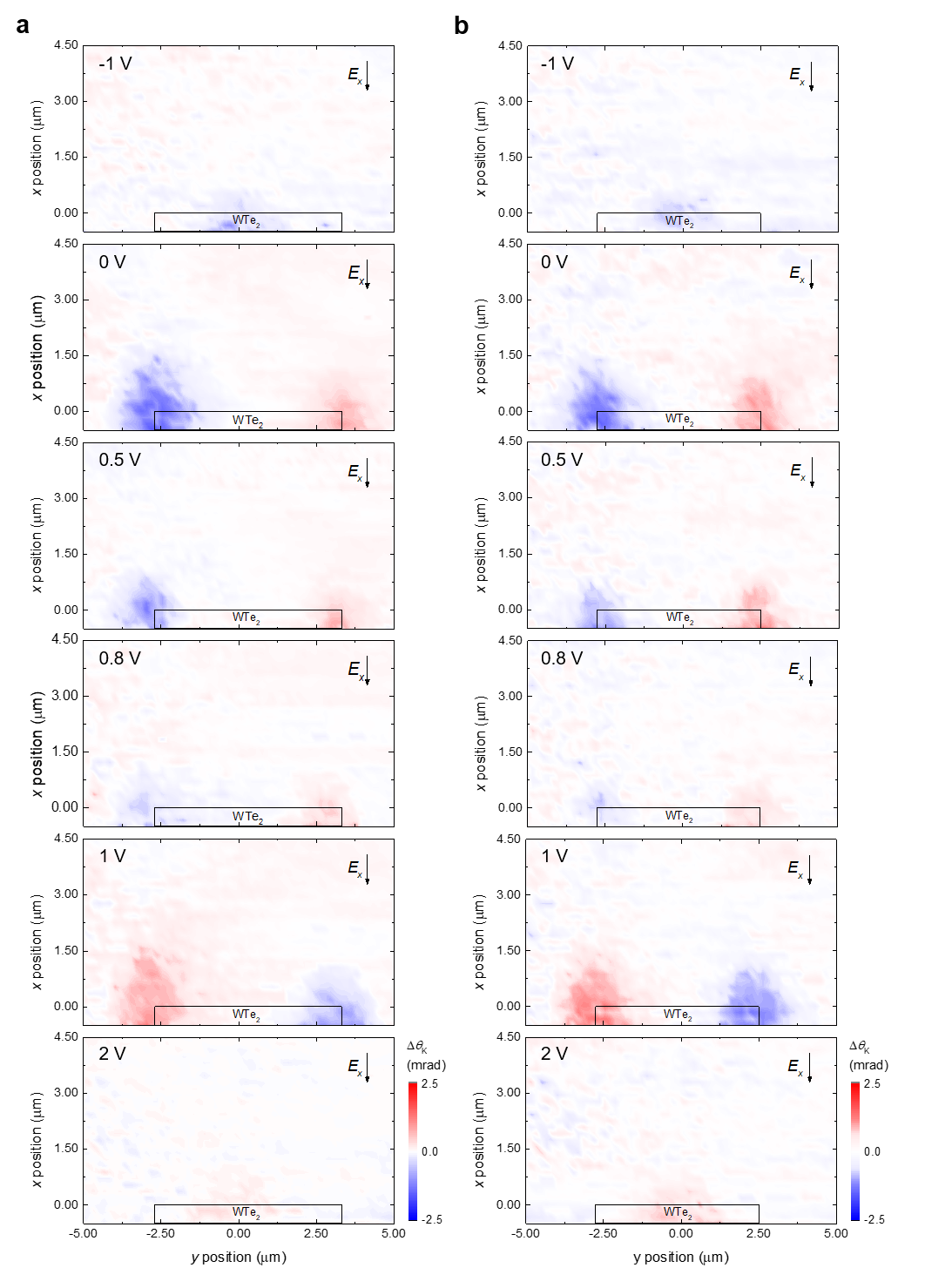


**Figure S13. Spatially resolved *V*_G_-dependent Δ*θ*_K_ measured in devices #2 and #3.** 2D contour plots of the *V*_G_-dependent Δ*θ*_K_ of device #2 (**a**) and #3 (**b**) are shown. An external magnetic field was not applied. The bias voltage of 0.5 V was applied to generate a longitudinal electric field in the -*x* direction. The applied *V*_G_ is indicated at the top left side of each contour plot. The black rectangle indicates the location of the top-end part of the multilayer WTe_2_. The localization of Δ*θ*_K_ and its sign change is identical to the result of device #1, as shown in Fig. S4.


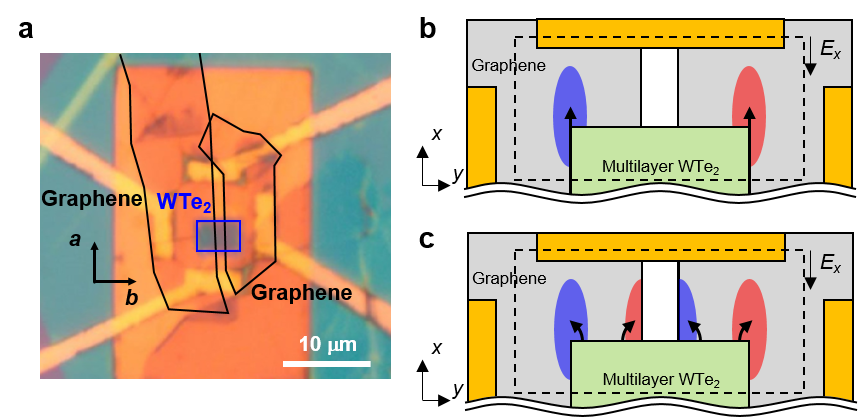


**Figure S14. An experiment proposed to isolate the hinge characteristics (device #4). a** An optical microscopy image is shown. Two monolayer graphene flakes are separated by a 1.5 μm gap. This device scheme is almost identical to the other devices other than the presence of a gap in graphene. The graphene layer for the electron transport measurement is located below WTe_2_. **b, c** Schematic representation for the expected Δ*θ*_K_ distribution when the spin-polarized electrons in graphene originate from the WTe_2_ hinge states (**b**) and when they originate from the WTe_2_ bulk (**c**). Dashed rectangles are the spatial windows that we performed the Kerr-rotation measurements. The black arrows are to represent electron transport.


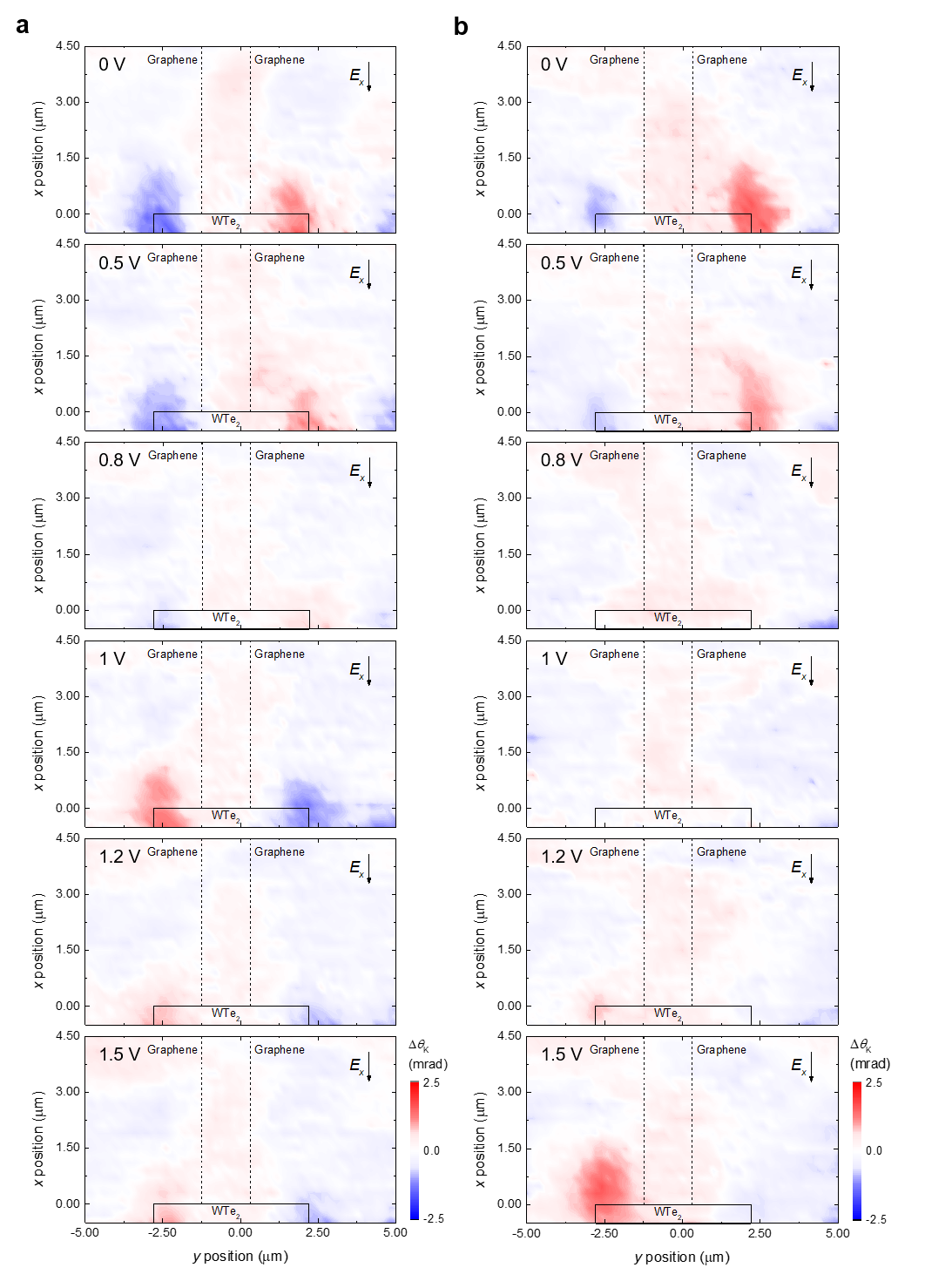


**Figure S15.** **The full dataset of spatially resolved *V*_G_-dependent Δ*θ*_K_ measurement (device #4).** 2D contour plots of the *V*_G_-dependent Δ*θ*_K_ from device #4 when *B_z_* = 0 (**a**) and *B_z_* = 1 T (**b**). Dashed lines indicate the edge of graphene flakes with the 1.5 μm gap between them. The black rectangle indicates the location of the top-end part of the multilayer WTe_2_. If the spin Hall contribution is the dominant origin of the observed Δ*θ*_K_, additional peaks and deeps should appear near the dashed line, as described in Fig. 4a. However, the distribution and *V*_G_ dependence of Δ*θ*_K_ are not affected by the existence of a graphene gap.


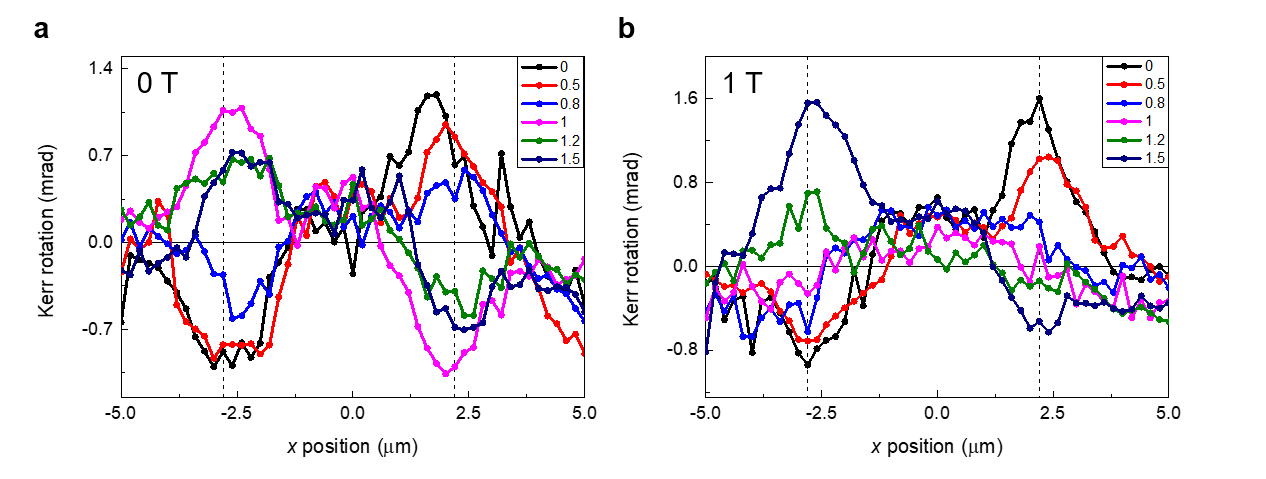


**Figure S16.** **Line-cut plot from the** **spatially resolved *V*_G_-dependent Δ*θ*_K_ measurement (device #4).** The line-cut plots are obtained from the 2D contour plots of Δ*θ*_K_ at y = 0.3 μm with varying *V*_G_ under *B_z_* = 0 T (**a**) and 1 T (**b**), which corresponds to Fig. S12a and Fig. S12b, respectively. Dashed lines indicate the x position of the WTe_2_ hinges in the real space. Similar to the result from device #1 (Fig. 3), suppression of Δ*θ*_K_ near the hinge due to the mass gap opening occurs when *V*_G_ = 0.8, 1 V.
